# Comparative Evaluation of Traditional and Controlled Drying Methods of Chestnuts (*Castanea sativa* Mill.): Impact on the Chemical Composition, Aromatic, and Sensory Profile of Flour

**DOI:** 10.3390/foods14111931

**Published:** 2025-05-29

**Authors:** Sofia Panzani, Francesca Venturi, Alessandro Bianchi, Pierina Díaz-Guerrero, Ylenia Pieracci, Guido Flamini, Isabella Taglieri, Chiara Sanmartin

**Affiliations:** 1 Department of Agriculture, Food and Environment, University of Pisa, Via del Borghetto 80, 56124 Pisa, Italy; sofia.panzani@phd.unipi.it (S.P.); francesca.venturi@unipi.it (F.V.); pierina.guerrero@phd.unipi.it (P.D.-G.); ylenia.pieracci@agr.unipi.it (Y.P.); isabella.taglieri@unipi.it (I.T.); chiara.sanmartin@unipi.it (C.S.); 2 Interdepartmental Research Centre “Nutraceuticals and Food for Health”, University of Pisa, Via del Borghetto 80, 56124 Pisa, Italy; guido.flamini@unipi.it; 3 Department of Pharmacy, University of Pisa, Via Bonanno Pisano 12, 56126 Pisa, Italy

**Keywords:** gluten-free flour, volatile organic compound, bioactive compounds, sensory analysis, forced-air drying, optimizing process

## Abstract

Chestnut flour, obtained through drying and milling of *Castanea sativa* fruits, has evolved from a subsistence food into a sought-after niche product, appreciated for its naturally gluten-free profile, high starch content, and richness in micronutrients. Over the past decade, its demand has steadily increased due to consumer perception of the health benefits associated with chestnut consumption. As the market for chestnut flour expanded from small-scale to large-scale production, alternative methods to the traditional process were developed. Its distinctive aroma and flavor are strongly influenced by processing methods, which are the focus of this study. Two drying approaches were compared: a traditional smoke-based method (drying house named *metato*) characterized by a wood-drying method and a controlled laboratory process using a forced-air dryer that maintained a constant temperature of 40 °C. The impact of these methods on the physico-chemical composition, volatile organic compounds (VOCs), and sensory properties of the flour was evaluated using chemical, instrumental, and sensory analyses. The traditional method enhanced the flour’s aromatic complexity and typicity through the application of smoke, which has been demonstrated to generate volatile organic compounds (VOCs), such as guaiacol, furfural, and o-creosol, that are associated with the smoked aroma. Nevertheless, if not properly managed, it can lead to undesirable sensory notes due to excessive smoke exposure. In contrast, the laboratory-controlled process ensured better preservation of bioactive compounds—such as polyphenols (351 mg GAE/100 g dm) and ascorbic acid (322 mg/kg dm)—while retaining the aroma notes associated with fresh chestnuts. Optimizing processing methods may support the valorization of chestnut flour as a high-quality ingredient in the modern gluten-free and functional food market.

## 1. Introduction

The chestnut tree (*Castanea* spp.), belonging to the Fagaceae family, is distributed across three main geographical areas and represented by four species: *Castanea sativa* Mill. in Europe, *Castanea crenata* Sieb. & Zucc. in Japan, *Castanea mollissima* Bl. in China and Korea, and *Castanea dentata* Borkh. in North America [1,2].

Among these, *Castanea sativa* Mill., native to Europe, Asia Minor, and North Africa, is the only species that grows wild in Europe and has been cultivated and valued since the time of the Roman Empire for its fruit and wood due to its adaptability to temperate climates [2,3,4]. Historically, chestnuts (*Castanea sativa* Mill.) have played a crucial role in the diets of many rural communities of mountainous marginal areas unsuitable for wheat cultivation [4,5].

Chestnuts are nutritionally rich, providing vitamins (B1, B2, C, E), minerals (Ca, P, K, Mg, S, Fe, Cu, Zn, Mn), dietary fiber, starch (~36% fresh weight), and phenolic compounds, while being low in fat (~3%) and cholesterol free [1,3,6,7,8,9]. However, their composition varies significantly based on cultivar and harvest year [6,10,11].

To extend their shelf life, given their high water content (~50%) [3], chestnuts were traditionally processed into flour [8,12], a naturally gluten-free product increasingly valued for both traditional and modern culinary applications [13,14,15,16,17].

Chestnut flour, being a nutritious, naturally gluten-free product, is particularly suited for consumers with gluten intolerance or sensitivity [6,13,14,15,16].

Italy counts fifteen chestnut-based products that have obtained the European Union’s Protected Designation of Origin (PDO) status. Among these, five are produced in the Tuscany region, including the renowned “*Farina di Neccio della Garfagnana PDO*” produced in the Garfagnana area and “*Farina di Castagne della Lunigiana PDO*” produced in the Lunigiana area [18].

Traditional chestnut flour production relies on the use of the *metato*, a two-level structure typically built from stone, lime and sand, where chestnuts are placed on upper racks made of chestnut sticks, or wire mesh in more recently restored structures immediately after harvest [19]. Drying is achieved through a slow, continuous fire maintained on the lower level, fueled exclusively with chestnut wood and kept steaming, covering it with residual peels from the previous year’s harvest [18].

This technique allows a gradual drying process, accompanied by mild smoking, without burning the fruit. As a result, fresh chestnuts are transformed into dried fruit with modified nutritional and aromatic properties, while moisture content is reduced to about 10% [20,21,22]. The dried chestnuts are then threshed to remove the outer shell (about 20% of the fruit), separate the edible nut, and finally ground in traditional mills using stone millstones, often water powered. The traditional drying process typically lasts a minimum of 40 days, and during the drying period, chestnuts are turned at least once to promote uniform moisture reduction.

Global chestnut production has increased steadily over the last decade due to consumers’ salient beliefs in the health benefits of chestnut consumption, and as the chestnut flour market has shifted from small to large scale, alternative methods to the traditional one have developed [8].

Industrial processes employ higher drying temperatures (40–80 °C) and mechanization, shortening drying times to as little as 48 h but potentially altering the nutritional and sensory profiles of the flour these are intended to ensure greater standardization, a feature which the traditional method currently lacks [12,22,23,24,25].

While traditional methods are valued for enhancing flavor, they often lack standardized process control, resulting in greater variability compared to modern controlled drying techniques.

The evolving demands of the agri-food sector in contemporary society are closely linked to ensuring human health safety and promoting sustainable development in production and processing methods [26].

While reviving traditional methods can be appealing, it is essential to assess how effectively these ancient practices align with modern expectations for product quality and health benefits. The traditional and industrial drying methods yield distinctly different products, as each technique alters the characteristics of the product in specific ways.

The quality of the chestnut dried in the “*metato*” might be harmed by the continuous change in temperature due to the manual managing of the wood fire [18]. Traditional drying could even lead to a high formation of toxic volatile compounds, such as furfural, guaiacol, and o-cresol. The latter two make the product taste smokey, or even burned if present in large amounts, and are formed precisely during the thermal degradation of lignin.

The traditional method, prized for imparting a distinctive smoky flavor to the product, should not be abandoned. However, it is essential to regulate temperature levels and maintain consistency throughout the process to ensure optimal quality. Clearly, improvements are needed in controlling the drying conditions inside the *metato*, as well as in managing the different stages of chestnut drying [22].

Moreover, due to climate change, the raw materials can vary significantly from year to year, for example showing lower moisture content, making it even more important to carefully monitor and adjust the drying process accordingly [27]. Moreover, the enhancement of the conventional technique would result in a reduction of waste, for instance, due to the development of mold and burns caused by inadequate drying management.

In this context, a critical evaluation of the impact of different processing methods, both traditional and non-traditional, on the chemical and sensory properties of chestnut flour becomes particularly relevant.

The present study provides a comparative evaluation of chestnut flours obtained using traditional drying techniques and a simulated industrial low-temperature process carried out under controlled laboratory conditions using a ventilated dryer. By characterizing chestnuts and the resulting flours using physical and chemical analyses as well as aromatic and descriptive sensory analysis, combined with the monitoring of processing conditions, this study aimed to evaluate the impact of different drying processes on the physicochemical and sensory properties of the final product.

## 2. Materials and Methods

### 2.1. Raw Materials and Production Process

The chestnuts and flour samples were provided by local growers of Garfagnana (Lucca, Italy) and coded as follows:

- M1t0: Fresh chestnuts
- M2t0: Fresh chestnuts
- FM1: Traditional flour obtained in *metato* 1 from M1t0 chestnuts
- FM2: Traditional flour obtained in *metato* 2 from M2t0 chestnuts
- FL: Laboratory-processed flour from M2t0 chestnuts

The chestnuts (*Castanea sativa* Mill.) used belong to the Carpinese, Pontecosi, and Rossola varieties. However, the percentage varietal composition of the different batches is not known.

Fresh chestnut samples were stabilized as soon as received. To ensure the preservation of the bioactive components, the samples were chopped (Monsieur Cuisine food processor, Silvercrest, Bad Wimpfen, Germany) and freeze-dried (LyoQuest lyophilizer, Azbil Telstar, S.L.U., Terrassa, Spain). The freeze-drying process lasted a total of 48 h, with a condenser temperature of −52.4 °C, a shelf temperature of 25 °C, and a pressure of 0.072 mBar.

The analyzed flours were produced using two distinct methods.

Samples FM1 and FM2 were produced using the traditional method. The two *metati* (drying houses), M1 and M2, were obtained from the municipality of Molazzana (Lucca, Italy), at the following coordinates:

− M1: 44.05939° N, 10.354385° E; approximately 800 m above sea level.
− M2: 44.066249° N, 10.371313° E; approximately 900 m above sea level.

The total drying time was different for the two *metati*. For M1, the drying process started on 16 October 2023 and ended on 29 November 2023, for a total of 44 days. In the case of M2, the process was started on 22 October 2023 and ended on 24 December 2023, for a total of 63 days.

Temperature and relative humidity trends (Figure S1a,b), within the traditional drying structures (*metati*), were continuously monitored using PKDLA1 data loggers (Parkside^®^, Neckarsulm, Germany). The devices were positioned on the top layer of chestnuts on each drying floor to ensure representative measurements of the environmental conditions affecting the drying process.

The FL flour was produced at the Food Science and Technology Laboratory (Department of Agriculture, Food and Environment, University of Pisa) simulating a small-scale industrial process using a Fruit Jerky Plus 6 dryer (Klarstein, Berlin, Germany). Fresh chestnuts from the same batch as M2t0 (later used for FM2 production) were dried at 40 °C with frequent weight monitoring to assess the drying progression for about 3 days (71 h; Figure S2).

After drying, the chestnuts were manually peeled (removal of both the outer shell and inner pellicle), chopped, and ground using a Monsieur Cuisine food processor (Silvercrest brand). Grinding was performed in ON/OFF cycles to avoid overheating. A temperature probe was used throughout the process to ensure that the product temperature never exceeded the drying temperature.

### 2.2. Chemical Analysis

#### 2.2.1. Determination of Dry Matter and Water Activity

The dry matter (dm) of samples was determined on about 5 g, dried at 105 °C until constant weight and expressed as percentage [28].

Water activity (a_w_) was measured [29] using a HygroPalm HP23-AW-A (Rotronic, Bassersdorf, Switzerland) hygrometer equipped with an HC2-AW probe, calibrated with the different range standards supplied with the instruments.

#### 2.2.2. Determination of Total Lipids

The total lipid (TL) content in the flour and fresh chestnut samples was determined using a Soxhlet extractor (SER148, Velp Scientifica Srl, Usmate Velate, Italy) following the methods previously reported [30]. Approximately 5 g of sample were placed in pre-dried and pre-weighed cellulose thimbles, which were previously dried in an oven at 105 °C. After drying, lipid extraction was carried out using *n*-hexane (ACS reagent, Sigma-Aldrich, Steinheim, Germany) as solvent. Following the extraction, the thimbles containing the de-oiled sample and the corresponding vessels containing the extracted oil were dried to remove any residual water and then weighed.

#### 2.2.3. Determination of Free Fatty Acidity

The determination of free fatty acidity (FFA) was conducted on 4 g of flour samples and chestnuts as previously reported [31]. Each sample was subjected to an extraction with 100 mL of a 50% hydroalcoholic solution neutralized with 0.1 N HCl (ACS reagent, Sigma-Aldrich, Steinheim, Germany) and maintained under magnetic stirring for 3 h.

Afterward, the sample was centrifuged at 966× *g* for 5 min, and a 50 mL aliquot of the supernatant was titrated with 0.02 N NaOH (ACS reagent, Sigma-Aldrich, Steinheim, Germany), using phenolphthalein as an indicator. By performing this procedure, the number of mL of 0.02 N alkali used for titration corresponds to the degree of acidity, which is referred to 100 parts of dry matter. The result was expressed as g oleic acid/100 g dm.

#### 2.2.4. Total Starch Sugars and Ascorbic Acid

Determinations of total starch, sugars, and ascorbic acid were performed using Megazyme enzyme kits (Megazyme^®^, Dublin, Ireland) following the instructions for each kit.

For the total starch content, the Megazyme^®^ K-TSHK was used, following the procedure described for samples containing resistant starch, simple sugars, and/or maltodextrins.

Glucose, fructose, and sucrose content were performed with the kit Megazyme^®^ K-SUFRG.

Ascorbic acid content was determined with Megazyme^®^ K-ASCO.

#### 2.2.5. Bioactive Compounds and Antioxidant Activity Analysis

In order to carry out the assays, a methanol extract was first prepared [32]. Briefly, 4 g were weighed in a Falcon tube, and 40 mL of 80% (*v*/*v*) methanol (Sigma-Aldrich, Steinheim, Germany) was added. The mixture was vortexed for approximately 15–20 s and subsequently subjected to sonication in an ultrasonic water bath for 30 min (temperature: 20 ± 2 °C, power: 100%, frequency: 40 kHz). After sonication, the samples were centrifuged at 10,000 rpm (10,733× *g*) for 10 min. The supernatant was filtered with a 0.45 μm cellulose acetate syringe filter. Extracts were stored at −20 °C for a minimum of 2 h before use in the assays. Hereafter, the methanolic extract is referred to as the “sample”.

The determination of total polyphenols content (TPC) was performed following the Folin–Ciocalteu method [33]. In 1 cm pathlength cuvettes, 100 μL of sample was added, followed by 1500 μL of 1:10 diluted Folin–Ciocalteu solution (Sigma-Aldrich, Steinheim, Germany) and 1875 μL of 7% (*w*/*v*) Na_2_CO_3_ solution (ACS reagent, Sigma-Aldrich, Steinheim, Germany). For the blank, the same volume of distilled water was used in place of the sample.

The cuvettes were then shaken and incubated in the dark for 1 h and 30 min. Finally, the absorbance was measured at 765 nm spectrophotometrically using a Cary 60 UV spectrophotometer (Agilent Technologies Inc., Santa Clara, CA, USA). The TPC was expressed as mg of gallic acid equivalents (GAE) per 100 g of dry matter (dm), based on calibration curve in the range 0–2 g/L of gallic acid (>98%, Sigma-Aldrich, Steinheim, Germany).

The determination of the total tannin content (TTC) was carried out according to the Bate–Smith method [34], based on the property of catechins, both monomeric and condensed, to oxidize in an acidic and alcoholic medium at high temperature (100 °C), releasing colored proanthocyanidins (Bate–Smith reaction). A volume of 2 mL of methanolic extract was placed in a test tube (A) with 6 mL of reaction mixture previously prepared as follows: 500 mL of 12 N HCl (ACS reagent, Sigma-Aldrich, Steinheim, Germany) + 500 mL of *n*-butanol (ACS reagent, Sigma-Aldrich, Steinheim, Germany) + 150 mg of Fe_2_(SO_4_)_3_ (ACS reagent, Sigma-Aldrich, Steinheim, Germany). The half of the mixture of the tube A was then transferred in glass test tube (B), closed with a screw cap and a Teflon seal, and placed in a hot oil bath (T = 105 °C) for 30 min. Then, the B tube was cooled at room temperature. At high temperatures, the acid hydrolysis of proanthocyanidins takes place.

Finally, the mixture in both A and B tubes was read spectrophotometrically at 550 nm with a 1 cm quartz cuvettes. The difference between the two readings was compared to the calibration curve in the range of 0–1 g/L of catechins (>98%, Sigma-Aldrich, Steinheim, Germany), allowing determination of the TTC value, expressed as mg of catechin equivalents (CE) per 100 g dm.

The antioxidant activity was assessed with three different assays: the ABTS method by reading the absorbance at 734 nm, the DPPH method by reading the absorbance at 515 nm, and the FRAP method by reading the absorbance at 593 nm [35]. The results were expressed as µmol Trolox equivalents (TE) per g dm of sample, based on different standard curves of Trolox (Sigma-Aldrich, Steinheim, Germany): 0–200 µmol L^−1^ for the DPPH assay, 0.2–1.5 mM range for ABTS, and 0–2.0 mM for the FRAP assay.

### 2.3. Color Determination

Color determination was performed using a tristimulus bench colorimeter (Eoptis^®^ CLM-196; Benchtop, Trento, Italy) with a white reference standard according to the CIE L* a* b* system (applying standard illuminant D65 with a 2° observer angle). The color of the samples was defined based on the three chromatic coordinates: lightness (L*), green-red (a*), and blue–yellow (b*) components.

The cylindric coordinates, namely, chroma (C*) and hue (h*); the white index (WI); and yellow index (YI) were calculated as previously reported [31] using the following equations:

(1) $$C^{*}=\sqrt{{\left(a^{*}\right)}^{2}+{\left(b^{*}\right)}^{2}}$$

(2) $$h^{*}=\arctan\;\left(\frac{b^{*}}{a^{*}}\right)$$

(3) $$\mathrm{WI}=100-\sqrt{{\left(100-L^{*}\right)}^{2}+{\left(a^{*}\right)}^{2}+{\left(b^{*}\right)}^{2}}$$

(4) $$YI=142.86\times \left(\frac{b^{*}}{L^{*}}\right)$$

The color difference between the samples was evaluated with the ΔE*_ab_ value calculated as previously reported [35] using the following equation:

(5) $${\Delta E}_{\mathrm{ab}}^{*}=\sqrt{\Delta {\left(L_{1}^{*}-L_{2}^{*}\right)}^{2}+\Delta {\left(a_{1}^{*}-a_{2}^{*}\right)}^{2}+\Delta {\left(b_{1}^{*}-b_{2}^{*}\right)}^{2}}$$

### 2.4. Volatile Organic Compound (VOC) Analysis

The aromatic profile of flours and fresh chestnuts were determined using the headspace solid-phase microextraction (HS-SPME) method, as reported by Pieracci et al. [36]. All samples were placed in glass vials, sealed with aluminum foil, and left to equilibrate for 30 min. After equilibration, a Supelco SPME fiber (100 µm, PDMS) (Supelco analytical, Bellefonte, PA, USA), conditioned according to the manufacturer’s guidelines, was exposed to the headspace for 30 min at room temperature. Then, the fiber was withdrawn into the needle and injected into a GC-MS system. The GC-EIMS (gas chromatography-electron impact mass spectrometry) analyses were conducted with an Agilent 7890B gas chromatograph (Agilent Technologies Inc., Santa Clara, CA, USA) equipped with an Agilent HP-5MS capillary column (30 m × 0.25 mm; coating thickness 0.25 mm) and an Agilent 5977B single quadrupole mass detector. The analytical conditions were set as follows: oven temperature ramp from 60 to 240 °C at 3 °C/min; injector temperature of 250 °C; transfer line temperature of 240 °C; helium used as the carrier gas at a flow rate of 1 mL/min. The analysis of the HS is performed with the splitless method. The acquisition parameters of the single quadrupole mass spectrometer were: full scan; scan range, 30–300 *m*/*z*; scan time, 1.0 s.

Peak identification was performed by comparing the retention times with those of authentic standards, evaluating their linear retention indices relative to a series of *n*-hydrocarbons (C8–C27), and matching the mass spectra against both commercial and laboratory-developed spectral libraries compiled from pure compounds, known essential oil components, and published MS data [37,38,39].

Odor description of VOCs was obtained from FLAVORNET [40] and The Good Scents Company Information System [41].

### 2.5. Sensory Evaluation

The flour sensory profile was evaluated using quantitative descriptive analysis (QDA) by a panel of eight trained judges (four females and four males, aged between 23 and 63 years) who are members of the “Experts Panel” of Department of Agriculture, Food and Environment of the University of Pisa. A sub-group of trained panelists participated in a consensus panel specifically designed generated descriptors and their definitions, prior to the tasting sessions. The research was conducted according to the ethical guidelines, and informed consent was obtained from all participants and obtained the approval of the Ethics Committee of the University of Pisa (protocol no. 0088081/2024).

**Figure 1:**
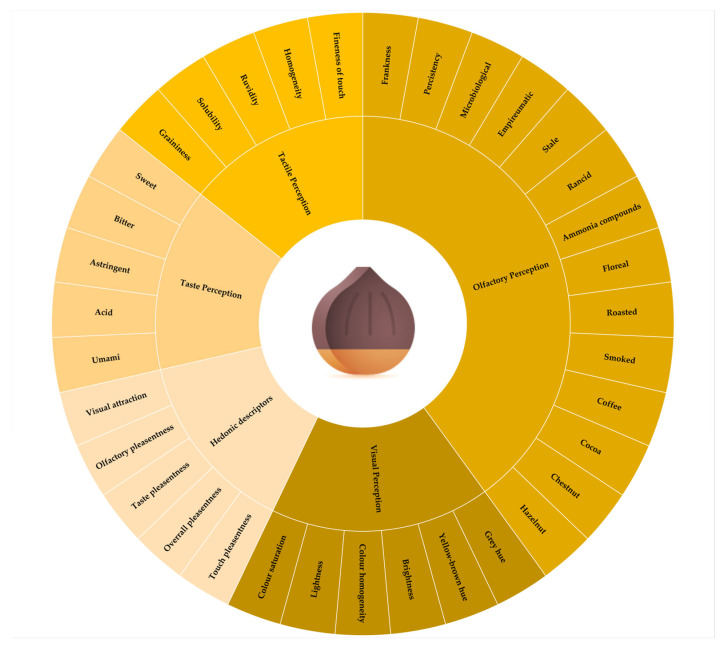
Descriptive parameters (quantitative and hedonic descriptors) used in the QDA test.

The QDA sensory test was conducted with a final set of 30 descriptive parameters (Figure 1) composed of both quantitative descriptors (sub-divided into visual, olfactory, tactile, and taste perceptions) and hedonic descriptors. In addition, judges were permitted to input free descriptors for each descriptor category.

Each judge assigned a score from 0 to 9 for each attribute (Figure S3), where 0 represents the absence of perception and 9 the maximum intensity, digitally acquired by the Input Sensory Soft 2.0 (ISS, Centro Studi Assaggiatori, Brescia, Italy). The samples were presented in a different order at each tasting session, and 5-minute intervals between each sample were set. Furthermore, a sample was randomly replicated to verify the performance of the panel at each tasting session

Finally, the overall hedonic index (HI), which represents the overall acceptability of the product, was calculated based on the mean of the hedonic parameters, which was converted to a scale from 0 to 10, as previously reported [42].

### 2.6. Statistical Analysis

Results were statistically analyzed with one-way analysis of variance (ANOVA) using CoStat version 6.451 software (CoHort Software, Pacific Grove, CA, USA) applying Tukey’s Honestly Significant Difference test (*p* < 0.05).

Sensory profile data were processed using Big Sensory Soft version 2.0 software (Centro Studi Assaggiatori, Brescia, Italy), and statistical analyses were performed by two-way interquartile ANOVA, with samples and panelists as main factors [43].

Finally, hierarchical cluster analysis (HCA), applying the Ward method and using two-way clustering, and other figures were performed using JMP Student version 18 software (SAS Institute, Cary, NC, USA).

## 3. Results and Discussion

### 3.1. Physico-Chemical Characterization

Table 1 reports the physico-chemical characterization of the analyzed chestnut and chestnut flour samples.

The two flours obtained using the traditional process (FM1 and FM2) showed slightly higher dry matter (dm) and lower water activity (a_w_) compared to the flour produced under controlled conditions (FL). Variability was also observed in the total lipid content (TL), with statistically significant difference among FL, FM2, and FM1. This may be attributed to the heterogeneity of the raw material (e.g., different varietal composition and harvest location).

The TL of fresh chestnuts (samples M1t0 and M2t0) may have been affected by the freeze-drying process used as a pre-treatment to stabilize the fresh sample; this process may have altered the structural characteristics, affecting the extraction yield of the lipids.

Lipid composition, along with dm content and a_w_, plays a critical role in determining the product’s shelf life.

Due to its low a_w_ and high dm content, chestnut flour is relatively resistant to microbial growth. However, although chestnut flour has a relatively low-fat content, its lipid fraction is characterized by a high percentage of unsaturated fatty acids (UFA), primarily linoleic acid (C18:2 *ω*6) and oleic acid (C18:1 *ω*9), as reported in the literature for different chestnut varieties [2,30,44]. UFAs are prone to oxidation, which can compromise the quality of the final product, especially under suboptimal storage conditions such as elevated temperature, light exposure, or inadequate atmospheric composition. The free fatty acid (FFA) value is a measure of lipid degradation. An increase in the value reflects a higher degree of lipid hydrolysis, leading to the availability of molecules more prone to subsequent oxidative reactions, which also impacts sensory quality. In the samples analyzed (Table 1), FFA values were very low. High levels of free fatty acids would negatively impact oxidative stability and, consequently, the product’s shelf life, increasing the risk of lipid rancidity with an impact on the sensorial quality. It is evident that elevated concentrations of free fatty acids can result in a decline in oxidative stability, thereby diminishing the product’s shelf life and heightening the probability of lipid rancidity. This phenomenon can also exert an influence on the sensorial quality of the product.

The data related to the carbohydrate fraction reveal an unexpected trend, particularly with respect to free sugars. Notably, sucrose (Table 2) exhibited a statistically significant increase after processing it into flour, contrary to expectations.

This phenomenon has also been observed by other research groups; however, to the best of our knowledge, no explanation can be provided at this time [6,45].

The data also show that during the transformation from fresh chestnuts (M1t0, M2t0) to flour (FL, FM1, FM2), the free sugar content undergoes a statistically significant increase due to the drying processes (Table 2). These processes, through water removal and heating of the raw material, cause the breakdown of part of the starch into simpler sugars.

The different sugar compositions could be a result of the varying compositions of the raw chestnut used, which may belong to different varieties [46]. As previously reported by Piccolo et al. [6], in a study concerning the varieties from the same geographical area, the different composition could impact the response to heat treatment.

Sugar transformation is affected by numerous variables, with heat treatment being the main factor [45,47], but the phytosanitary condition of chestnuts also plays a role.

The time and conditions of storage prior to heat treatment, mainly temperature and humidity, are also critical factors [48,49]. If these conditions are inadequate, they may induce physicochemical changes in the fruit, including the degradation of carbohydrate reserves necessary for cellular respiration, a process that begins when the fruit naturally falls and continues until enzymatic activity and water activity allow germination to occur [3].

**Figure 2:**
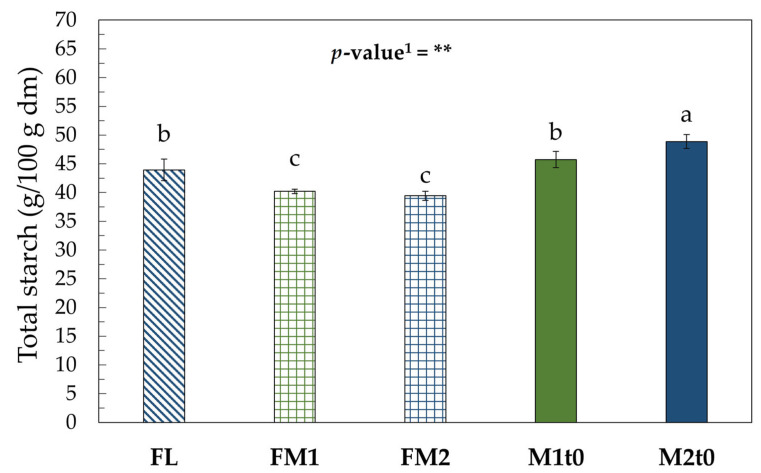
Total starch (g/100 g dm). Values represent the mean *±* standard deviation (SD) of three samples. Different letters indicate significant difference among values (Turkey’s HSD, *p* < 0.05). ^1^ Significance level: ** = *p* < 0.01.

Figure 2 shows the total starch content of fresh chestnuts and their derived flours. Although significant decreases in starch content are observed when comparing M1t0 and M2t0 with their corresponding flours (M1t0: FM1; M2t0: FM2, FL), these differences are not substantial enough to account for the increase in free sugar content reported in the flours (Table 1).

Correia et al. [50] report that the starch granules of *C. sativa* are composed of type C crystalline units, a mixture of type A and type B structures. Correlating with the findings of Wang et al. [51], who reports that during freeze-drying there is a modification of the starch component of *C. mollissima*, it can be hypothesized that the freeze-drying treatment applied to the chestnuts for analysis may have altered their components. Freeze-drying may influence the type B crystals, which are destroyed due to the removal of the bound water located between the helices.

Buléon et al. [52] reported that the B-type structure is composed of repeated maltoside units; consequently, breaking the bonds between the helical chains leads to the release of maltodextrins (linear chains of D-glucose units ranging from 2 to 20). Following the removal of internal bound water, if the length of the resulting chains falls within the range of maltodextrins, these could have been eliminated during the starch extraction process used for the enzymatic kit, which includes pre-washing steps aimed at removing free sugars and maltodextrins that would interfere with the quantification of total starch.

### 3.2. Bioactive Compounds and Antioxidant Activity

**Figure 3:**
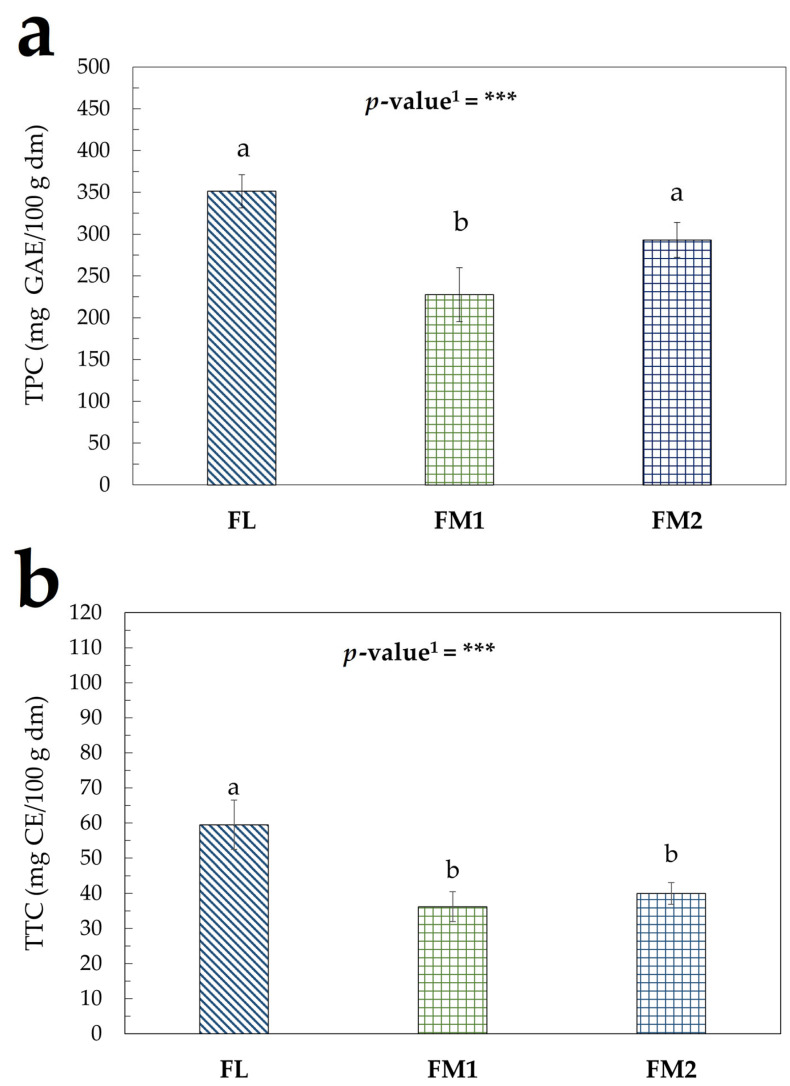
Bioactive compounds in flour samples: (**a**) TPC (mg GAE/100 g dm); (**b**) TTC (mg CE/100 g dm). Values represent the mean ± standard deviation (SD) of three samples. Different letters indicate significant difference among values (Turkey’s HSD, *p* < 0.05). ^1^ Significance level: *** = *p* < 0.001.

The trend of TPC and TTC in the flours samples is shown in Figure 3a,b, which highlights the presence of significant differences.

The differences observed between the samples of chestnut flour can be attributed to several factors. The FL sample underwent a shorter heat treatment at controlled temperatures, which favored rapid dehydration of the product (Figure S2). This may help to preserve the phenolic compounds present in the raw chestnuts, which are more likely to degrade in traditionally produced flours that were exposed to heat for longer periods under less controlled conditions (Figure S1a,b). These observations are consistent with findings reported by Conti et al. [22].

**Figure 4:**
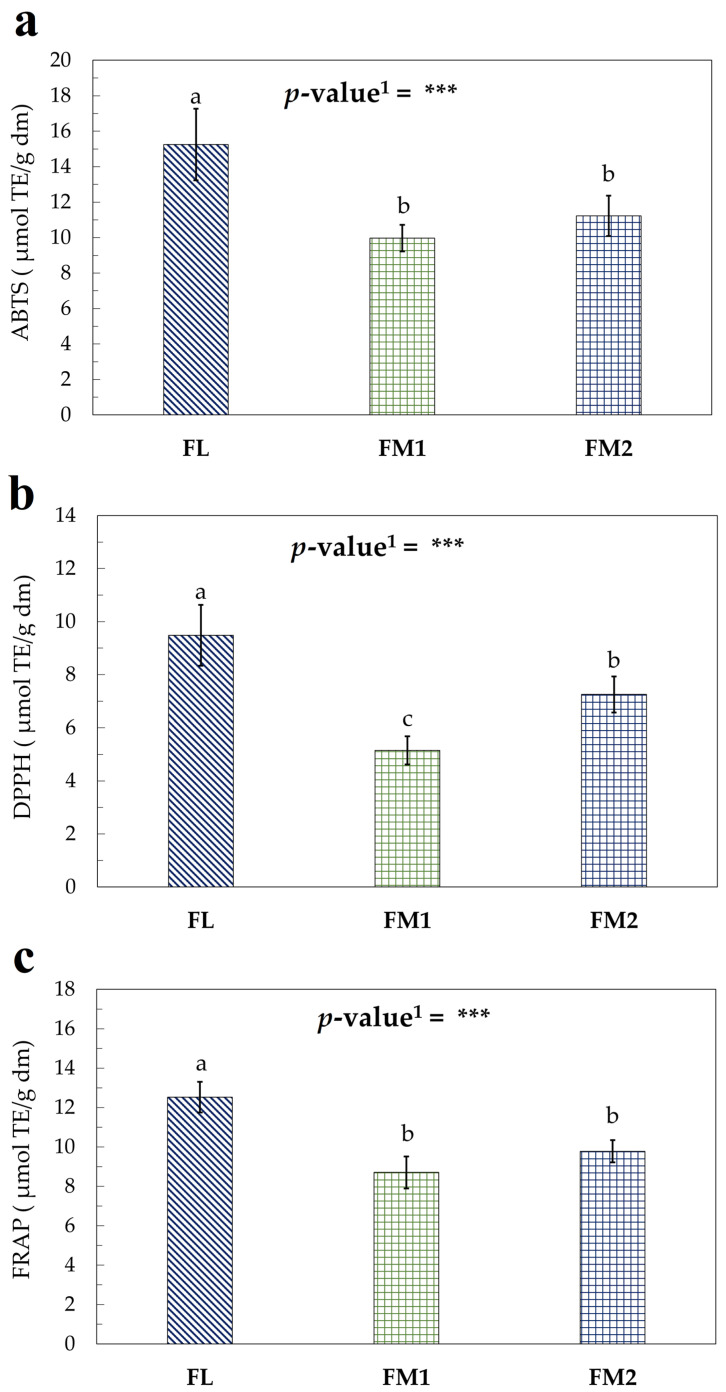
Antioxidant activity assay of flour samples expressed as μmol TE/g dm: (**a**) ABTS; (**b**) DPPH; (**c**) FRAP. Values represent the mean *±* standard deviation (SD) of three samples. Different letters indicate significant difference among values (Turkey’s HSD, *p* < 0.05). ^1^ Significance level: *** = *p* < 0.001.

The assays performed to evaluate the antioxidant activity of the flour samples (Figure 4a–c) produced results consistent with the trends observed in the TPC and TTC assays. These findings confirm that the processing conditions adopted for the FL sample led to reduced degradation of bioactive compounds, further supporting the hypothesis that shorter and more controlled heat treatments help preserve the nutritional and functional properties of chestnut flour [15,53].

**Figure 5:**
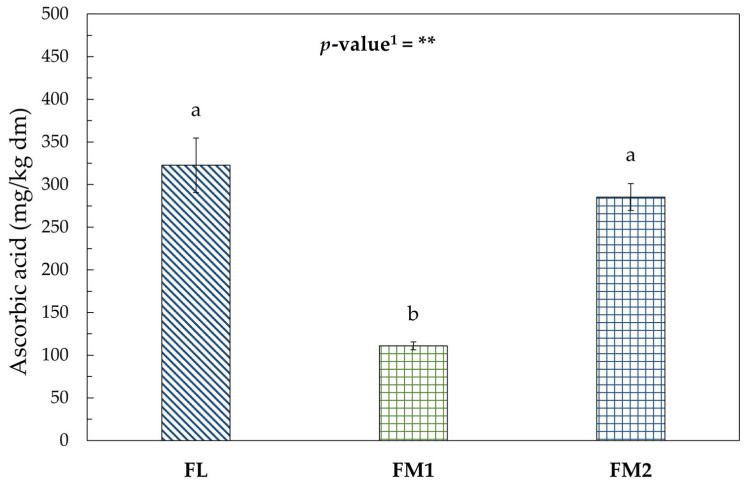
Ascorbic acid (mg/kg dm) in flour samples. Values represent the mean *±* standard deviation (SD) of three samples. Different letters indicate significant difference among values (Turkey’s HSD, *p* < 0.05). ^1^ Significance level: ** = *p* < 0.01.

This trend could also be correlated with the behavior of ascorbic acid as reported in Figure 5.

The behavior of chestnut bioactive compounds should also be considered in relation to the different chestnut varieties, which respond differently [6].

In this case (FM1 and FM2 comparison), the composition of the batch used for flour production was not defined in terms of the proportion of the different varieties, which could have influenced the results.

Moreover, the reduced processing time combined with constant temperature conditions likely contributed to limiting the degradation of thermolabile compounds.

As reported in the literature, ascorbic acid is highly sensitive to thermal treatments. According to Nguyen [54], lower drying temperatures and shorter drying times favor better retention of vitamin C in dried chestnuts. The vitamin C content remained comparable in FL and in FM2, both derived from the same batch of chestnuts.

The FM1 sample, despite undergoing a shorter drying process than FM2, showed a significantly lower ascorbic acid concentration, likely due to compositional differences in the raw material. Barros et al. [55] reported that the different behavior of ascorbic acid during processing could also be influenced by variation in shell and peel properties, which can alter thermal transfer and the diffusivity coefficient of the chestnut.

It can therefore be concluded that the drying process conducted under controlled laboratory conditions and stable temperatures—ensuring shorter exposure times to heat and minimizing thermal fluctuations—resulted in better preservation of the bioactive compounds (Figures S1a,b and S2).

The temperature trend inside the *metati*, as illustrated in Figure S1a,b, fluctuated between approximately 5 °C and 35 °C, causing an instability during the drying process. Instead, relative humidity (%) varied between approximately 30% and 90%. For the M1 and M2 *metati*, the weighted averages of temperature (°C) and relative humidity (%) were 19.0 °C and 81.5% as well as 17.4 °C and 71.2%, respectively. These factors were probably affected by external environmental conditions, unlike the laboratory dryer where these variables are kept constant over time allowing for more constant dehydration (Figure S2).

### 3.3. Color Evaluation

The color of chestnuts changes during the processing into flour. Undoubtedly, the compounds present in the raw fruit undergo modifications due to spontaneous reactions.

The results of the colorimetric evaluation are reported in Table 3. The results show an increase in lightness (L*) from raw chestnuts (M1t0, M2t0) to flour samples (FL, FM1, FM2), accompanied by an increase in the white index (WI) and a decrease in the yellow index (YI), following the thermal degradation and the oxidative phenomena that affecting the carotenoids [56,57].

The calculation of ΔE*_ab_ (Table 4) provides values representing the color differences between the flour samples.

In this case, the analyzed samples showed only slight differences, but these fell into two distinct ranges. Specifically, the difference between the FL sample and the FM1 sample falls within the ΔE*_ab_ below 2.5, indicating a slight but perceptible color difference [58].

On the other hand, the ΔE*_ab_ between the FL and FL2 samples, obtained from the same batch of chestnuts, shows a value of 3.83, indicating a noticeable but distinguishable color variation [31].

Since both flours were produced from the same batch of chestnuts, the differences can mainly be attributed to the different processing methods applied.

### 3.4. Volatile Organic Compound (VOC) Profile

The complete chemical composition of the volatile emissions of the samples analyzed is reported in Table 5.

The GC-MS analysis permitted the identification of a total of 62 compounds, accounting for 95.0–99.7% of the whole volatile profiles. The identified chemical constituents mainly belonged to the chemical classes of monoterpene hydrocarbons and non-terpenes derivatives. In particular, monoterpene hydrocarbons were detected in high percentages (47.2%) in the laboratory-processed flour (FL), mainly characterized by γ-terpinene (20.4%) and myrcene (13.6%), responsible for woody and spicy notes, respectively. Nevertheless, the volatile profile of FL flour also showed noteworthy relative amounts of the non-terpenic acids (18.8%), alcohols (15.2%), and aldehydes (12.8%), with acetic acid, 1,3- and 2,3-butanediol, and nonanal being the chief compounds, respectively.

Acids, alcohols, and aldehydes also represented the main chemical classes of the volatile emission of the samples FM1 and FM2. Acetic acid was the only compound belonging to the class of non-terpenic acids and was detected at the highest relative abundance in the FM1 flour (32.0%), although FM2 (21.2%) and FL (18.8%) also showed high percentages. The presence of acetic acid could be due to bacteria and yeasts typically present on fresh raw materials, as demonstrated by studies conducted on other products [59]. The higher presence of this molecule in the FM1 sample may be related to the post-harvest conditions of the fresh chestnuts before processing. As these conditions are not standardized, significant differences among samples may occur.

Among non-terpenic alcohols, the 1,3- and 2,3-butanediol isomers represented important components of the volatile emission of FM1 and FM2 as well, and their presence could be also related to microbial metabolism, particularly to yeast activity [60,61]. Nevertheless, the traditional flour *metato* 2 exhibited remarkable relative amounts of 3-ethyl-1-hexanol, associated with floral scent notes, which were not detected in any of the other samples. Finally, regarding aldehydes, although considerable percentages of nonanal were also found in FM1 and FM2, these samples were particularly rich in hexanal (10.6 and 8.8%, respectively) responsible for green aroma notes.

Analyzing the data, it was observed that the aromatic complexity of the flours (FL, FM1, and FM2) particularly differ from that of the fresh chestnuts (M1t0 and M2t0), probably as the result of the thermal treatment and the traditional smoking process applied to traditional flours. In detail, both fresh chestnut samples were characterized by alcohols as the most abundant chemical class. However, while M1t0 showed isopentyl alcohol as the main compound of this class, accounting for 13.0%, M2t0 exhibited 2,3-butanediol (33.8%) followed by 1,3-butanediol (22.9%) as major constituents.

Interestingly, the volatile emission of fresh chestnuts did not show the presence of aldehydes, which instead have been characterized in significant percentages, were greatly found in the flours, as previously reported. Indeed, aldehyde compounds, as evidenced in the literature by various research groups [15,20], can derive from the drying process, as these compounds were not found in the fresh chestnut samples (M1t0 and M2t0). The temperatures used both in the traditional drying process and the laboratory processes, although not high, could be sufficient to start lipid peroxidation, leading to the formation of aldehydes and ketones. In our case, compounds like hexanal, heptanal, octanal, (*E*)-2-octenal, nonanal, and decanal were detected, which might originate from the degradation of unsaturated lipids present in the chestnuts [3,15,20,21]. Nevertheless, fresh chestnuts spontaneously emitted remarkable percentages of ketones, with great differences between the samples analyzed. Indeed, in addition to showing higher relative amounts of the buttery scented acetoin, which in turn was the only ketone detected in M2t0, M1t0 also featured not negligible percentages of 2-pentanone and 2-heptanone. Fresh chestnuts *metato* 1 (M1t0) headspace, different from that of *metato* 2 (M2t0), was also characterized by relevant amounts of phenolic compounds, mainly represented by 3-phenylpropanol (20.2%), responsible for spicy and floral notes, and p-ethylguaiacol (4.6%), associated with smoky flavors [20,21]. Considerable percentages of phenols were detected in the volatile emission of the flours, even though, in this case, they were mainly represented by o-guaiacol, creosol, and phenol, found in higher relative quantities in FM2 than in FM1. The presence of these compounds could be related to the degradation of lignin into simpler phenolic compounds during the flour thermal treatment and smoking process of the traditional method, in agreement with Cantini et al. [19]. Thermal treatment is also related to the presence of furanic compounds that can originate from the degradation and the rearrangement of carbohydrates via the Maillard reaction [62,63]. Among these compounds, furfural, furfuryl alcohol, 2-acetylfuran, and 2-pentylfuran were detected exclusively in the flours. Conversely, 2,3-dihydrobenzofuran was found in low percentages in M0t2 [20,22].

Hierarchical cluster analysis (HCA) graphically summarized the above discussed differences in the volatile emissions of the analyzed samples (Figure S4). Indeed, the obtained dendrogram was constituted by two first-level clusters, dividing fresh chestnuts from the flours. In turn, the traditional flours FM1 and FM2 constituted a sub-cluster by themselves, within the upper cluster also comprising the sample FL, highlighting greatest similarities in their volatile emissions.

### 3.5. Sensory Profile

**Figure 6:**
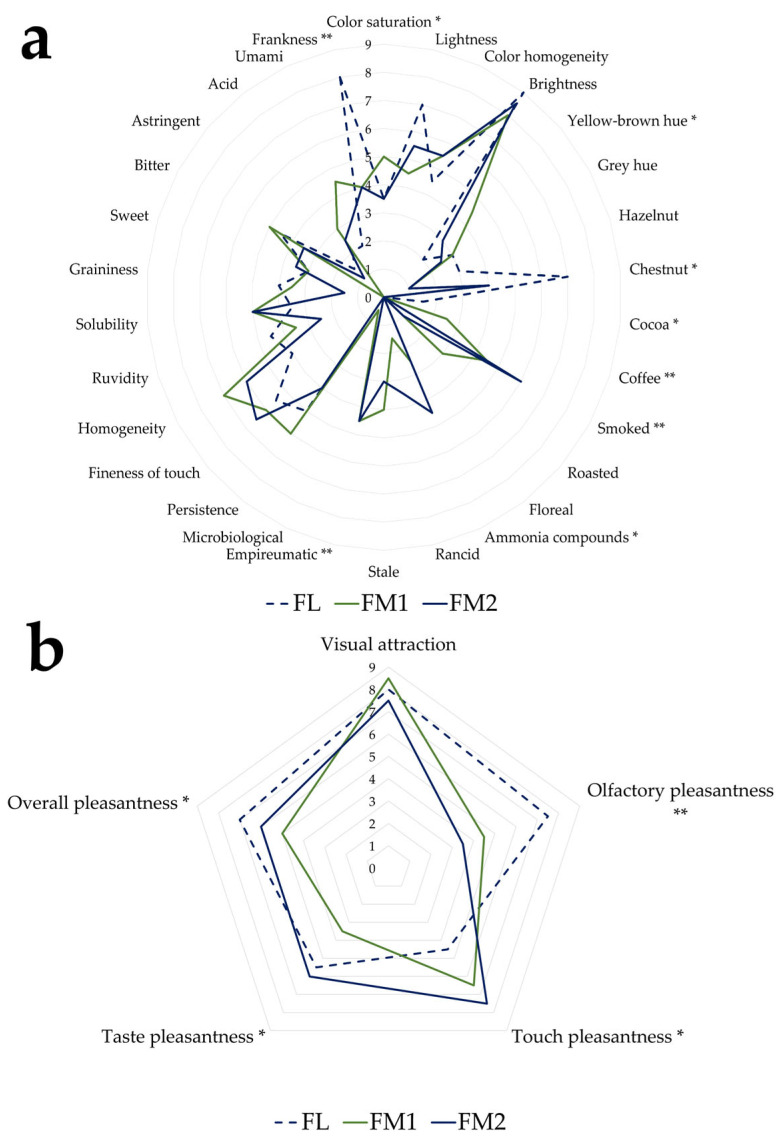
Median of the descriptor of the sensory profile of chestnut flour: (**a**) quantitative; (**b**) hedonic significance level: * = *p <* 0.05; ** = *p* < 0.01; without asterisk = *p* ≥ 0.05.

The sensory analysis conducted on the three chestnut flour samples permitted the characterization of their respective sensory profiles. Figure 6a,b compares the profiles obtained using descriptive parameters from the sensory evaluation. The descriptors for which statistically significant differences were observed among the samples are marked with an asterisk.

The main differences were observed between sample FL and samples FM1 and FM2. As expected, based on colorimetric data, the samples exhibited significant visual differences in terms of color saturation and yellow–brown hues. From an olfactory perspective, sample FL, which was not subjected to smoking, showed a value of zero for the empyreumatic descriptor, unlike the other two samples.

Furthermore, sample FL displayed a higher score for the chestnut descriptor, whereas FM1 and FM2 were more strongly characterized by smoky, coffee, and cocoa notes (Figure 6a), typically associated with traditional smoking processes [20]. These results are also consistent with the values of VOCs such as o-guaiacol, furfural, and creosol, which are associated with odors like smoky and baked, as reported in Table 5 and present in samples FM1 and FM2.

Regarding frankness (absence of defects), sample FL also reported higher positive values than the other samples.

Sample FM2 exhibited higher intensity in the smoky and bitter descriptors (Figure 6a), likely attributable to the extended duration of the traditional drying process and prolonged exposure to thermal degradation phenomena. In contrast, sample FL, processed under controlled conditions, displayed a more delicate profile characterized by sweet and fruity notes, suggesting better preservation of the original aromatic components.

**Figure 7:**
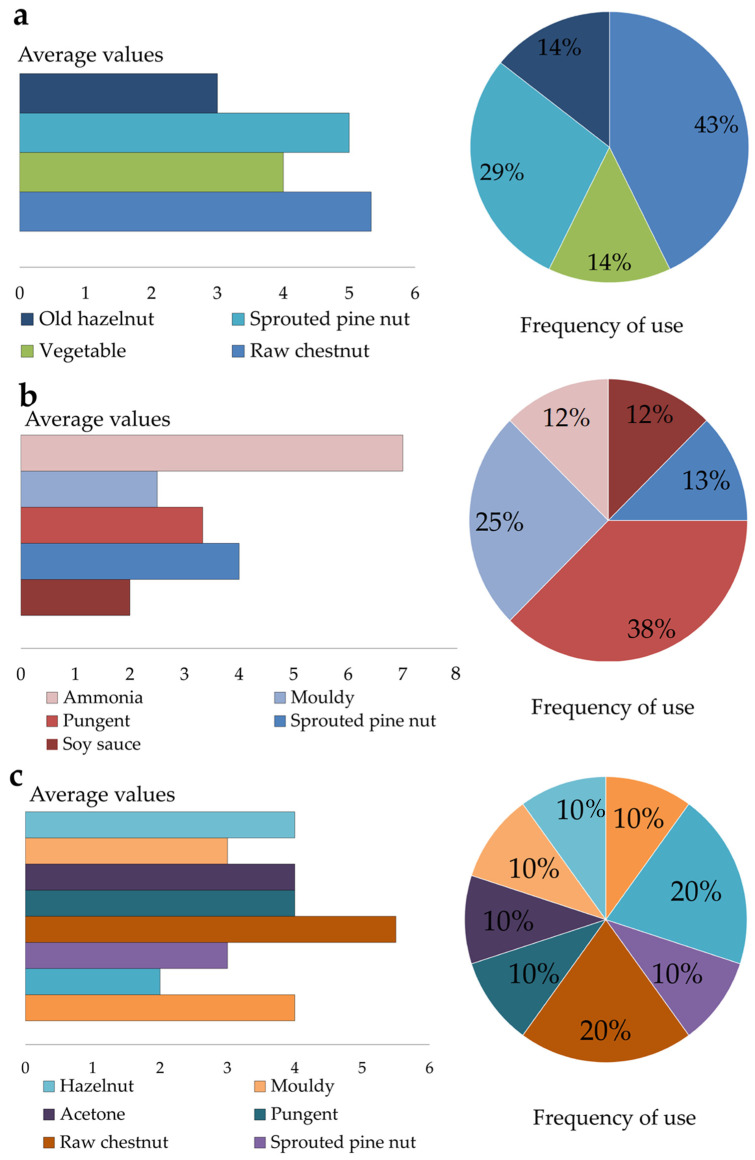
Average of free descriptors and their frequency of use for each flour sample: (**a**) FL; (**b**) FM1; (**c**) FM2.

In addition, the panel evaluated the free descriptors associated with the olfactory profile of the samples, as illustrated in Figure 7a–c. The graphs report the average values of the free attribute and the frequency of their use.

In sample FL (Figure 7a), descriptors related to fruity and vegetal families were identified, with the predominant descriptor being raw chestnut. In contrast, samples FM1 (Figure 7b) and FM2 (Figure 7c) were predominantly characterized by descriptors associated with the empyreumatic category (e.g., pungent, metallic) most likely as a result of the traditional smoking process, which was not applied to sample FL. Sample FM2 also exhibited greater aromatic complexity compared to the other samples, a finding that is consistent with the volatile compound (VOC) analysis previously discussed. Both FM1 and FM2 flours were produced using traditional smoking techniques for 43 and 66 days, respectively; however, sample FM2 underwent a markedly prolonged smoking treatment, which may have led to excessive exposure to smoke and contributed to its distinct aromatic profile.

Concerning the hedonic descriptors, FM1 and FM2 received the lowest scores for olfactory pleasantness, which negatively affected their overall evaluation. In contrast, FL flour obtained the highest ratings for both olfactory and overall pleasantness, although it scored lowest for tactile pleasantness (Figure 6b).

To summarize, the highest hedonic index (HI) was achieved by the sample FL (7.2), followed by FM2 (6.7), while FM1 recorded the lowest value (6.2) (Figure S5).

These results are consistent with the instrumental analysis of volatile compounds and confirm that different processing methods significantly affect the sensory perception of the final product. Sensory analysis thus proved to be an essential tool for integrating and completing the qualitative evaluation of the flours under investigation.

## 4. Conclusions

Traditional chestnut flour is a product obtained from the drying and milling of fruits from various cultivars of *Castanea sativa*. Historically, this foodstuff has been of fundamental importance as a staple food.

Today, it is considered a niche product, increasingly valued for its distinctive sensory properties and its notable nutritional qualities, being rich in carbohydrates, minerals, and vitamins, and naturally gluten free.

The present study aimed to evaluate the impact of traditional and controlled processing methods on the quality of chestnut flour, representing a preliminary investigation into the main critical aspects of chestnut flour production. To this end, a chemical-physical and volatile and sensory characterization was performed on both fresh chestnuts and the flours obtained from them.

The laboratory-scale drying process, conducted at controlled temperatures over shorter times, allowed better preservation of bioactive compounds, as noted for the total phenolic content and antioxidant activity, and overall compositional integrity. In addition, the primary smell descriptors associated with the fresh raw material were also retained. Traditional processing, while imparting a distinctive aromatic profile due to smoke exposure during drying, proved to be less controllable compared to laboratory-scale drying using a ventilated system (stable at 40 °C), and the process is highly affected by weather conditions, as evident from the observed temperature range, which spans from 5 °C to 35 °C. Although this traditional method has the potential to enrich the flour with complex and unique aromatic notes, inadequate control of its duration or intensity may lead to an excessively marked empyreumatic profile, due to the presence of some VOCs, like as o-guaiacol, furfural, and creosol, which, if present in excessively high concentrations, could result in a less pleasant or unbalanced sensory perception.

To enhance the quality of traditionally produced flour, it is crucial to gain a deeper understanding of the drying phase. The adoption of technologies capable of monitoring the key parameters (e.g., temperature, humidity, and drying time) could minimize the formation of undesirable compounds while safeguarding the product’s traditional sensory identity, helping manufacturers to understand when the drying process needs to be remodeled and when the optimum level of drying has been reached. Such technological advancements would not only optimize the nutritional and sensory quality of the flour but would also contribute to more efficient and sustainable production practices, particularly for producers aiming to scale up without compromising product excellence.

As this is a preliminary study, future research will focus on a broader nutritional characterization of chestnut flour, including protein and mineral content, to deepen the understanding of how different drying methods influence its quality. These insights will support the development of optimized drying protocols that preserve the nutritional and sensory attributes of the final product while maintaining the identity and authenticity of traditional processing.

## Figures and Tables

**Table 1:** Physico-chemical characterization chestnut and chestnut flour samples.

Parameter	Units	*p*-Value ^1^	FL	FM1	FM2	M1t0	M2t0
Dry matter (dm)	%	***	89.10 ± 0.09 ^b^	91.72 ± 0.07 ^a^	91.40 ± 0.05 ^a^	53.56 ± 0.20 ^c^	49.56 ± 0.13 ^d^
a_w_		ns	0.50 ± 0.07	0.41 ± 0.01	0.43 ± 0.02	n.d.	n.d.
TL	g/100 g dm	***	4.14 ± 0.01 ^a^	2.13 ± 0.01 ^b^	4.50 ± 0.01 ^a^	1.30 ± 0.01 ^c^	1.58 ± 0.01 ^bc^
FFA	g oleic acid/100 g dm	***	0.13 ± 0.01 ^a^	0.14 ± 0.01 ^a^	0.14 ± 0.01 ^a^	0.07 ± 0.01 ^b^	0.10 ± 0.01 ^b^

The values represent the mean (±SD). Different letters in each row correspond to statistically different values (Turkey’s HSD, *p* < 0.05). ^1^ Significant level: ns = *p* ≥ 0.05; *** = *p* < 0.001. n.d. = not detected.

**Table 2:** Sucrose, D-glucose, and D-fructose content expressed as g/100 g of dm.

Parameters	Units	*p*-Value ^1^	FL	FM1	FM2	M1t0	M2t0
Sucrose	g/100 g dm	***	19.04 ± 2.14 ^a^	21.57 ± 0.51 ^a^	15.93 ± 0.69 ^b^	9.88 ± 1.26 ^c^	9.31 ± 0.33 ^c^
D-Glucose	g/100 g dm	***	0.70 ± 0.04 ^c^	1.70 ± 0.11 ^a^	0.61 ± 0.06 ^c^	0.91 ± 0.09 ^b^	0.75 ± 0.06 ^bc^
D-Fructose	g/100 g dm	***	0.58 ± 0.03 ^b^	1.44 ± 0.04 ^a^	0.48 ± 0.03 ^c^	0.44 ± 0.03 ^c^	0.40 ± 0.04 ^c^

The values represent the mean (±SD). Different letters in each row correspond to statistically different values (Turkey’s HSD, *p* < 0.05). ^1^ Significant level: *** = *p* < 0.001.

**Table 3:** Color parameters of different samples.

Parameter	*p*-Value ^1^	FL	FM1	FM2	M1t0	M2t0
L*	***	88.43 ± 0.84 ^a^	84.14 ± 1.23 ^b^	87.14 ± 0.97 ^a^	68.66 ± 0.81 ^c^	71.19 ± 0.84 ^c^
a*	***	0.25 ± 0.03 ^d^	1.26 ± 0.05 ^a^	0.67 ± 0.03 ^c^	0.98 ± 0.06 ^b^	0.98 ± 0.06 ^b^
b*	***	11.86 ± 0.72 ^c^	14.13 ± 0.52 ^b^	12.80 ± 0.11 ^bc^	22.32 ± 0.79 ^a^	22.32 ± 0.79 ^a^
C*	***	11.86 ± 0.73 ^c^	12.56 ± 1.91 ^b^	13.41 ± 1.52 ^bc^	22.34 ± 0.79 ^a^	22.34 ± 0.79 ^a^
h*	***	1.55 ± 0.01 ^a^	1.48 ± 0.01 ^c^	1.51 ± 0.04 ^b^	1.53 ± 0.01 ^b^	1.53 ± 0.01 ^b^
WI	***	83.41 ± 0.40 ^a^	78.72 ± 1.20 ^b^	79.86 ± 2.87 ^a^	61.51 ± 0.56 ^d^	63.54 ± 0.58 ^c^
YI	***	19.16 ± 1.03 ^c^	24.01 ± 1.18 ^b^	22.54 ± 3.14 ^bc^	46.44 ± 1.67 ^a^	44.79 ± 1.61 ^a^

Values are presented as the mean *±* standard deviation (SD) of three samples. In the same row, different letters indicate significant difference among values (Turkey’s HSD, *p* < 0.05). ^1^ Significance level: *** = *p* < 0.001.

**Table 4:** Color differences (ΔE*_ab_) among the different flour samples.

ΔE*_ab_	FL	FM1	FM2
**FL**		2.32	3.83
**FM1**			1.55
**FM2**			

**Table 5:** Composition of the VOC emissions (relative abundance (%)) of the fresh chestnuts and derived flours.

Compound	l.r.i ^2^	Odor	*p*-Value ^1^	Relative Abundance (%)				
				FL	FM1	FM2	M1t0	M2t0
*Monoterpene hydrocarbons*								
α-Pinene	941	Balsamic	***	0.8 ± 0.02 ^a^	- ^3,b^	- ^b^	- ^b^	- ^b^
Sabinene	977	Woody	***	1.6 ± 0.03 ^a^	- ^b^	- ^b^	- ^b^	- ^b^
β-Pinene	982	Herbal	***	3.2 ± 0.06 ^a^	- ^b^	- ^b^	- ^b^	- ^b^
Myrcene	993	Spicy	***	13.6 ± 0.53 ^a^	- ^b^	- ^b^	- ^b^	- ^b^
δ-3-Carene	1012	Citrus	***	2.8 ± 0.34 ^a^	- ^b^	- ^b^	- ^b^	- ^b^
*p*-Cymene	1028	Citrus	***	3.2 ± 0.06 ^a^	- ^b^	- ^b^	- ^b^	- ^b^
Limonene	1032	Citrus	***	- ^c^	2.0 ± 0.06 ^a^	- ^c^	- ^c^	0.1 ± 0 ^b^
γ-Terpinene	1062	Woody	***	20.4 ± 0.81 ^a^	- ^b^	- ^b^	- ^b^	- ^b^
Terpinolene	1090	Herbal	***	1.6 ± 0.03 ^a^	- ^b^	- ^b^	- ^b^	- ^b^
*Alcohols*								
Isobutyl alcohol	627	Musty	*	- ^b^	- ^b^	- ^b^	1.2 ± 0.05 ^ab^	2.3 ± 1.92 ^a^
1-Butanol	657	Fusel	***	- ^b^	- ^b^	- ^b^	0.2 ± 0.01 ^a^	- ^b^
Isopentyl alcohol	736	Musty	***	- ^c^	- ^c^	- ^c^	13 ± 0.45 ^a^	6.5 ± 2.47 ^b^
2-Methylbutanol	737	Fusel	***	- ^b^	- ^b^	- ^b^	6.3 ± 0.39 ^a^	5.3 ± 2.32 ^a^
1-Pentanol	766	Balsamic	***	- ^c^	0.9 ± 0.01 ^a^	0.4 ± 0.2 ^b^	- ^c^	- ^c^
1,3-Butanediol	788		***	5.6 ± 0.11 ^bc^	7.7 ± 0.1 ^b^	2.7 ± 0.06 ^c^	5.6 ± 2.74 ^bc^	22.9 ± 1.1 ^a^
2,3-Butanediol	789		***	4.8 ± 0.1 ^bc^	9.9 ± 0.27 ^b^	4 ± 0.01 ^c^	8.9 ± 3.64 ^bc^	33.8 ± 2.38 ^a^
Furfuryl alcohol	858	Burning	***	- ^b^	- ^b^	1 ± 0.02 ^a^	- ^b^	- ^b^
1-Hexanol	871	Herbal	***	2.8 ± 0.34 ^b^	2.2 ± 0.19 ^b^	1.1 ± 0.08 ^cd^	5.6 ± 0.93 ^a^	0.2 ± 0 ^d^
1-Heptanol	970	Herbal	***	- ^b^	- ^b^	- ^b^	0.4 ± 0.13 ^a^	0.1 ± 0 ^b^
1-Octen-3-ol	982	Musty	***	- ^b^	1 ± 0.11 ^a^	1.1 ± 0.08 ^a^	- ^b^	- ^b^
3-Ethyl-1-hexanol	1031	Floral	***	- ^b^	- ^b^	8.8 ± 0.41 ^a^	- ^b^	- ^b^
1-Octanol	1071	Herbal	***	- ^c^	0.7 ± 0.06 ^a^	- ^c^	0.2 ± 0.01 ^b^	- ^c^
Phenylethyl alcohol	1111	Floral	***	2 ± 0.36 ^b^	2.1 ± 0.09 ^b^	0.7 ± 0.09 ^c^	3.7 ± 0.39 ^a^	2.7 ± 0.3 ^b^
*Ethers*								
2-Acetylfuran	913	Fruity	***	- ^b^	- ^b^	0.6 ± 0.01 ^a^	- ^b^	- ^b^
2-Pentyl furan	992	Fruity	***	- ^b^	3.5 ± 0.69 ^a^	- ^b^	- ^b^	- ^b^
γ-Caprolactone	1056	Herbal	***	- ^b^	0.8 ± 0.01 ^a^	- ^b^	- ^b^	- ^b^
2,3-Dihydrobenzofuran	1221	Harsh	***	- ^b^	- ^b^	- ^b^	- ^b^	0.3 ± 0.05 ^a^
*Phenols*								
Phenol	983		***	- ^d^	1.6 ± 0.01 ^b^	4.5 ± 0.1 ^a^	0.5 ± 0.25 ^c^	0.1 ± 0 ^d^
*o*-Cresol	1057	Musty	***	- ^c^	- ^c^	3.9 ± 0.3 ^a^	0.9 ± 0.27 ^b^	- ^c^
*p*-Cresol	1078	Floral	***	- ^c^	- ^c^	1.3 ± 0.13 ^a^	0.4 ± 0.13 ^b^	- ^c^
*o*-Guaiacol	1091	Smoky	***	- ^d^	2.6 ± 0.07 ^b^	14.0 ± 0.02 ^a^	1.6 ± 0.76 ^c^	0.1 ± 0 ^d^
Veratrole	1149	Musty	***	- ^b^	0.7 ± 0.01 ^a^	- ^b^	- ^b^	- ^b^
Creosol	1193	Smoky	***	- ^c^	1.2 ± 0.15 ^b^	4.9 ± 0.09 ^a^	- ^c^	- ^c^
3-Phenylpropanol	1232	Spicy, floral	***	- ^b^	- ^b^	- ^b^	20.2 ± 3.41 ^a^	- ^b^
*p*-Ethylguaiacol	1280	Smoky	**	- ^b^	- ^b^	- ^b^	4.6 ± 1.92 ^a^	- ^b^
*p*-Vinylguaiacol	1314	Spicy		- ^b^	- ^b^	- ^b^	- ^b^	1.1 ± 0.1 ^a^
*Esters*								
Ethyl acetate	611	Fruity	**	- ^b^	1 ± 0.06 ^b^	0.7 ± 0.12 ^b^	0.6 ± 0.09 ^b^	4.5 ± 1.98 ^a^
Isopentyl acetate	876	Fruity	***	- ^b^	- ^b^	- ^b^	- ^b^	0.7 ± 0.05 ^a^
Butyrolactone	914		***	4.8 ± 0.1 ^a^	2.8 ± 0.12 ^b^	1.4 ± 0.03 ^c^	- ^d^	- ^d^
Ethyl hexanoate	998	Fruity	***	- ^b^	- ^b^	- ^b^	- ^b^	0.1 ± 0.00 ^a^
Ethyl 2-phenylacetate	1246	Fruity, honey	***	- ^b^	- ^b^	- ^b^	- ^b^	0.1 ± 0.00 ^a^
2-Phenylethyl acetate	1259	Fruity, floral	***	- ^b^	- ^b^	- ^b^	- ^b^	0.1 ± 0.00 ^a^
*Aldehydes*								
Hexanal	802	Herbal	***	3.6 ± 0.33 ^c^	10.6 ± 0.09 ^a^	8.8 ± 0.61 ^b^	- ^d^	- ^d^
Furfural	839	Baked	***	- ^c^	2.6 ± 0.04 ^a^	1.8 ± 0.16 ^b^	- ^c^	- ^c^
Heptanal	901	Herbal	***	- ^c^	0.8 ± 0.01 ^a^	0.5 ± 0.09 ^b^	- ^c^	- ^c^
Octanal	1001		***	2.4 ± 0.05 ^b^	3 ± 0.19 ^a^	1.4 ± 0.03 ^c^	- ^d^	- ^d^
5-Ethylcyclopent-1-enecarboxaldehyde	1035		***	- ^b^	0.4 ± 0 ^a^	- ^b^	- ^b^	- ^b^
*(E)*-2-Octenal	1063	Fruity	***	- ^b^	0.6 ± 0 ^a^	0.7 ± 0.12 ^a^	- ^b^	- ^b^
Nonanal	1102	Fruity	***	4.8 ± 0.1 ^a^	4.9 ± 0.29 ^a^	2.9 ± 0.34 ^b^	- ^c^	- ^c^
Decanal	1204	Fruity	***	1.2 ± 0.38 ^a^	- ^b^	- ^b^	- ^b^	- ^b^
*(E,E)*-2,4-Nonadienal	1215	Herbal	***	0.8 ± 0.02 ^a^	- ^b^	- ^b^	- ^b^	- ^b^
*Ketones*								
2-Pentanone	696	Fruity	***	- ^b^	- ^b^	- ^b^	2.8 ± 0.12 ^a^	- ^b^
Acetoin	709	Buttery	***	- ^c^	- ^c^	- ^c^	17.8 ± 3.36 ^a^	8.1 ± 3.17 ^b^
2-Heptanone	894	Fruity/herbal	***	- ^c^	0.6 ± 0 ^b^	0.5 ± 0.09 ^b^	2.7 ± 0.23 ^b^	- ^c^
6-Methyl-5-hepten-2-one	987	Herbal	***	- ^b^	- ^b^	- ^b^	0.5 ± 0.02 ^a^	- ^b^
3-Octen-2-one ^4^	1042	Spicy, herbal	***	- ^c^	0.9 ± 0.05 ^a^	0.6 ± 0.01 ^b^	- ^c^	- ^c^
2-Nonanone	1093	Herbal	***	- ^b^	- ^b^	- ^b^	1.5 ± 0.18 ^a^	- ^b^
*Acids*								
Acetic acid	599	Acidic	***	18.8 ± 0.78 ^b^	32.0 ± 0.99 ^a^	21.2 ± 1.35 ^b^	- ^d^	9.3 ± 3.96 ^c^
*Other non-terpene derivatives*								
Styrene	896	Balsamic/plastic	*	- ^b^	- ^b^	- ^b^	- ^b^	1.2 ± 0.91 ^a^
*n*-Undecane	1100		***	- ^b^	- ^b^	0.4 ± 0.01 ^a^	- ^b^	- ^b^
Naphthalene	1181	Pungent	***	- ^c^	2.5 ± 0.02 ^b^	4 ± 0.01 ^a^	- ^c^	- ^c^
*n*-Dodecane	1200		***	0.8 ± 0.02 ^a^	- ^b^	0.8 ± 0.02 ^a^	- ^b^	- ^b^
**Chemical classes**								
*Terpenes*								
Monoterpene hydrocarbons			***	47.2 ± 0.15 ^a^	2.00 ± 0.06 ^b^	- ^c^	- ^c^	0.1 ± 0 ^c^
Non-terpene derivatives								
Alcohols/ethers/phenols			***	15.2 ± 0.49 ^d^	34.9 ± 0.89 ^c^	49.1 ± 0.53 ^b^	73.3 ± 2.74 ^a^	75.3 ± 3.68 ^a^
Esters			**	4.8 ± 0.1 ^a^	3.8 ± 0.18 ^ab^	2.2 ± 0.15 ^bc^	0.6 ± 0.09 ^c^	5.5 ± 2.03 ^a^
Aldehydes/ketones			***	12.8 ± 0.54 ^bc^	24.5 ± 0.06 ^a^	17.3 ± 0.10 ^b^	25.2 ± 2.81 ^a^	8.1 ± 3.17 ^c^
Acids			***	18.8 ± 0.78 ^b^	32.0 ± 0.99 ^a^	21.2 ± 1.35 ^b^	- ^d^	9.3 ± 3.96 ^c^
Others			**	0.8 ± 0.02 ^c^	2.5 ± 0.02 ^b^	5.2 ± 0.02 ^a^	- ^d^	1.2 ± 0.91 ^c^
**Total identified**				**99.6 ± 0.01**	**99.7 ± 0.1**	**95 ± 0.55**	**99 ± 0.16**	**99.6 ± 0.05**

Values are presented as the mean *±* standard deviation (SD) of three samples. The superscript lowercase letters in the row indicate statistical differences among the samples (Turkey’s HSD, *p* < 0.05). ^1^ Significance level: *** *p* < 0.001, ** *p* < 0.01, * *p* < 0.05; ^2^ Linear retention indices on a HP5-MS capillary column; ^3^ Not detected; ^4^ Correct isomer not determined.

## Data Availability

The original contributions presented in the study are included in the article/Supplementary Materials; further inquiries can be directed to the corresponding author.

## Supplementary Materials

The following supporting information can be downloaded at: https://www.mdpi.com/article/10.3390/foods14111931/s1. Figure S1: Trend of the temperature (°C) and relative humidity (%): (a) inside the *metato* 1; (b) inside the *metato* 2. Figure S2: Weight loss in the drying of the FL sample. Figure S3: Sensory sheet used by the judges on the ISS portal. Q = quantitative descriptors; H = Hedonic descriptor. Figure S4: HCA of VOCs. Figure S5: Hedonic index (HI) of the chestnut flour samples. Different letters indicate significant differences among values (Turkey’s HSD, *p* < 0.05).
