# Supplementary material for: Comparative Evaluation of Traditional and Controlled Drying Methods of Chestnuts (Castanea sativa Mill.): Impact on the Chemical Composition, Aromatic, and Sensory Profile of Flour
Source: Foods. 2025 May 29;14(11):1931. doi: 10.3390/foods14111931 (PMC12155469; doi:10.3390/foods14111931)
Supplement: Supplementary file 1 [file foods-14-01931-s001.zip › foods-3649057-supplementary.pdf]

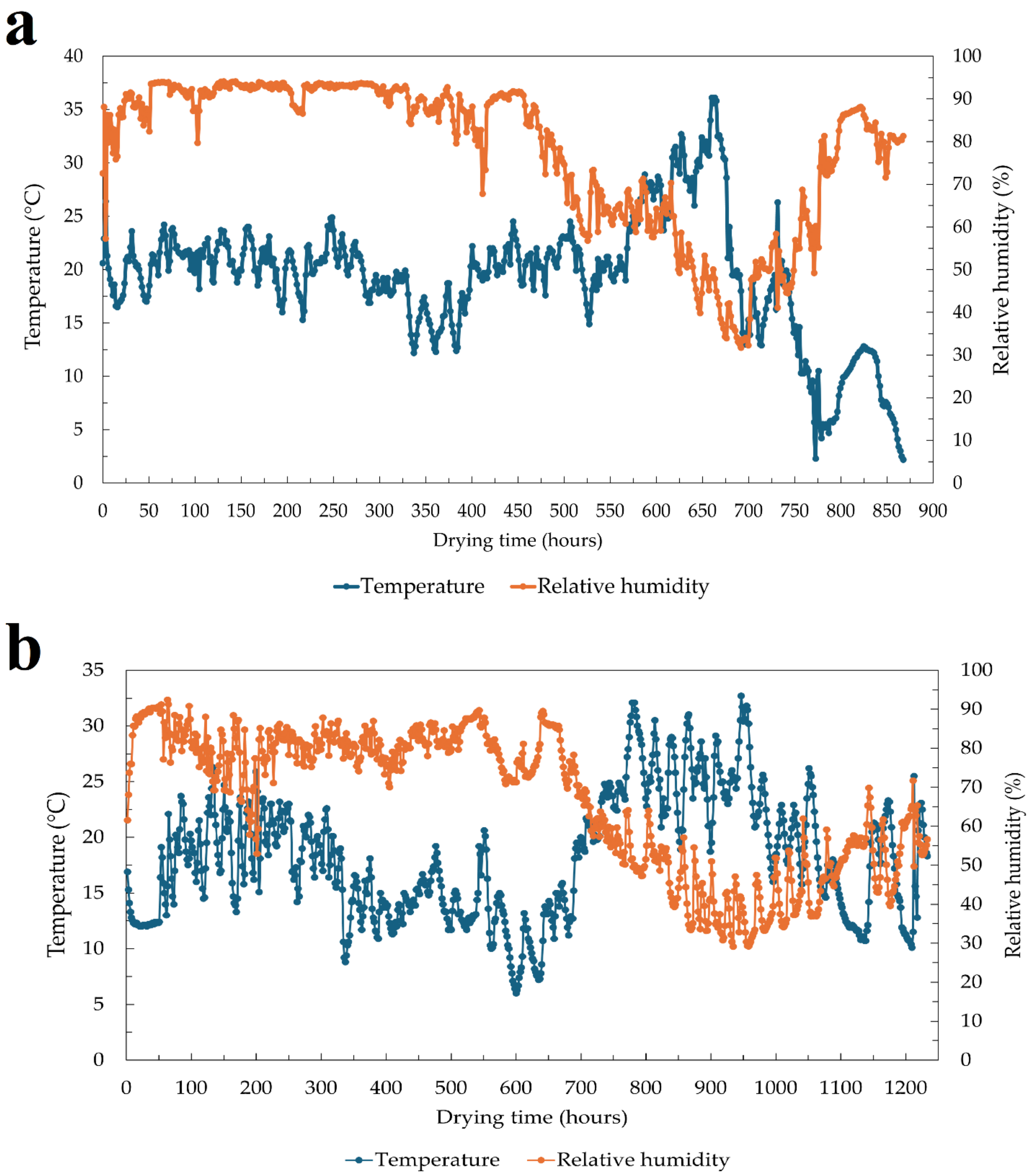

Figure S1. Trend of the temperature (°C) and relative humidity (%): (**a**) inside the *metato* 1; (**b**) inside the *metato* 2.

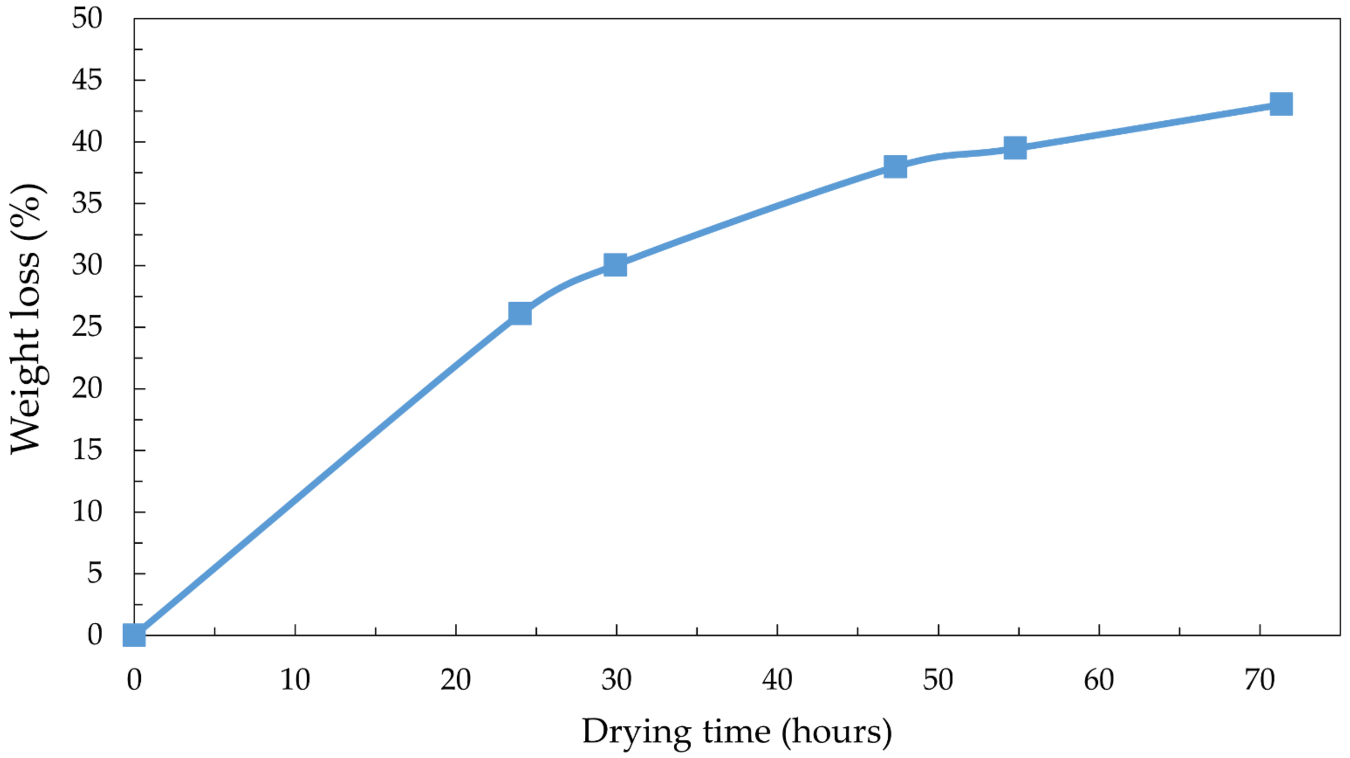

Figure S2. Weight loss in the drying for the FL sample.

Chestnut Flour		
**Descriptor type**	**Descriptor**	**0 - 9**
**Visual perception**		
Q	Colour saturation	□ □ □ □ □ □ □ □ □ □
Q	Lightness	□ □ □ □ □ □ □ □ □ □
Q	Homogeneity	□ □ □ □ □ □ □ □ □ □
Q	Brightness	□ □ □ □ □ □ □ □ □ □
Q	Yellow-brown hue	□ □ □ □ □ □ □ □ □ □
Q	Grey hue	□ □ □ □ □ □ □ □ □ □
H	**Visual Attraction**	□ □ □ □ □ □ □ □ □ □
**Olfactory perception**		
Q	Hazelnut	□ □ □ □ □ □ □ □ □ □
Q	Chestnut	□ □ □ □ □ □ □ □ □ □
Q	Cocoa	□ □ □ □ □ □ □ □ □ □
Q	Coffee	□ □ □ □ □ □ □ □ □ □
Q	Smoked	□ □ □ □ □ □ □ □ □ □
Q	Roasted	□ □ □ □ □ □ □ □ □ □
Q	Floreal	□ □ □ □ □ □ □ □ □ □
Q	Ammonia compounds	□ □ □ □ □ □ □ □ □ □
Q	Rancid	□ □ □ □ □ □ □ □ □ □
Q	Stale	□ □ □ □ □ □ □ □ □ □
Q	Empireumatic	□ □ □ □ □ □ □ □ □ □
Q	Microbiological	□ □ □ □ □ □ □ □ □ □
Q	Persistency	□ □ □ □ □ □ □ □ □ □
Q	Frankness	□ □ □ □ □ □ □ □ □ □
H	**Olfactory Pleasantness**	□ □ □ □ □ □ □ □ □ □
**Tactil perception**		
Q	Fineness of touch	□ □ □ □ □ □ □ □ □ □
Q	Homogeneity	□ □ □ □ □ □ □ □ □ □
Q	Ruvidity	□ □ □ □ □ □ □ □ □ □
Q	Solubility	□ □ □ □ □ □ □ □ □ □
Q	Graininess	□ □ □ □ □ □ □ □ □ □
H	**Touch Pleasantness**	□ □ □ □ □ □ □ □ □ □
**Taste perception**		
Q	Sweet	□ □ □ □ □ □ □ □ □ □
Q	Bitter	□ □ □ □ □ □ □ □ □ □
Q	Astringent	□ □ □ □ □ □ □ □ □ □
Q	Acid	□ □ □ □ □ □ □ □ □ □
Q	Umami	□ □ □ □ □ □ □ □ □ □
H	**Taste Pleasantness**	□ □ □ □ □ □ □ □ □ □
H	**Overall Pleasantness**	□ □ □ □ □ □ □ □ □ □

Figure S3. Sensory sheet used by the judges on the ISS portal. Q = quantitative descriptors; H = Hedonic descriptor.

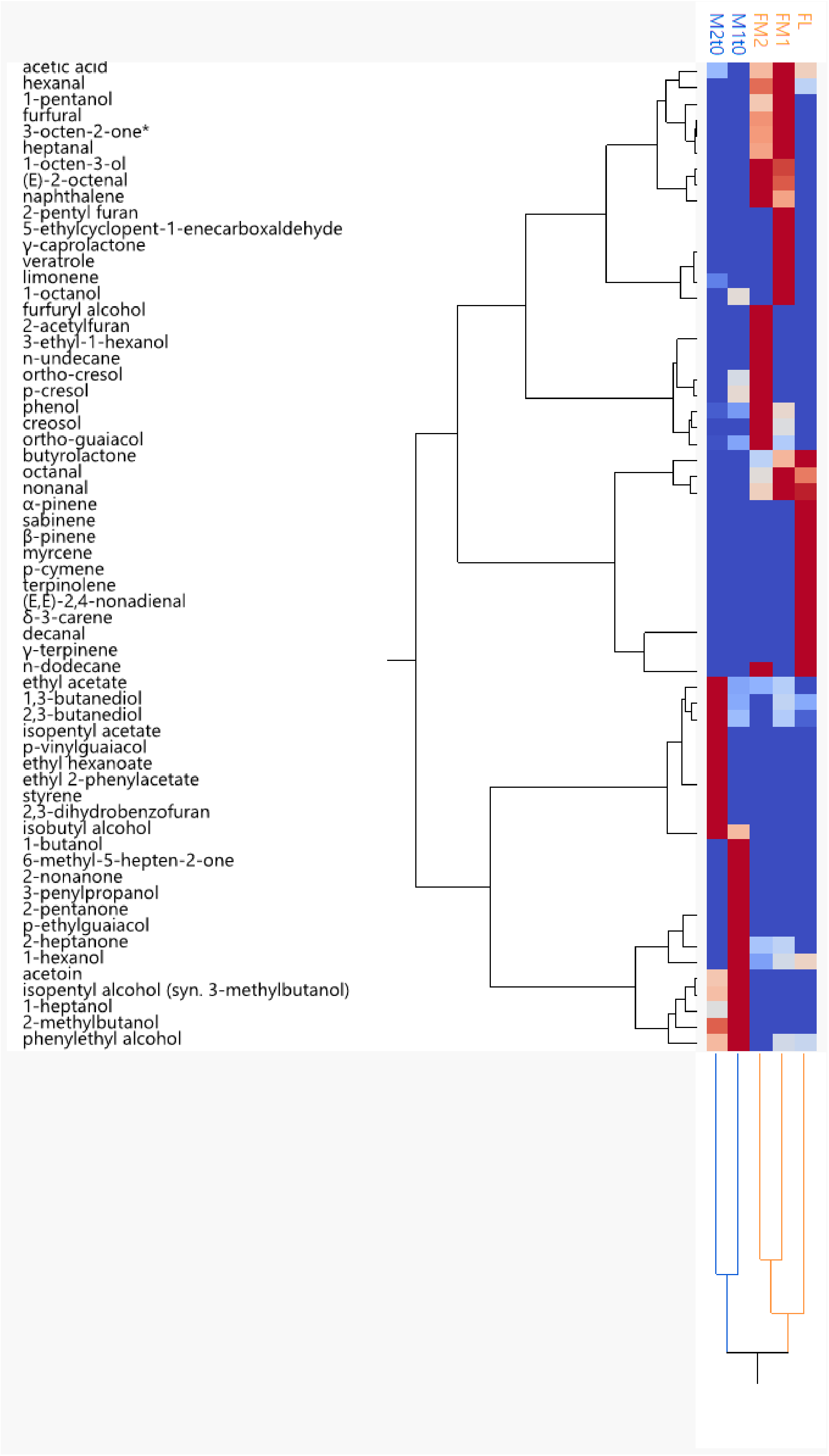

Figure S4. HCA of VOCs .

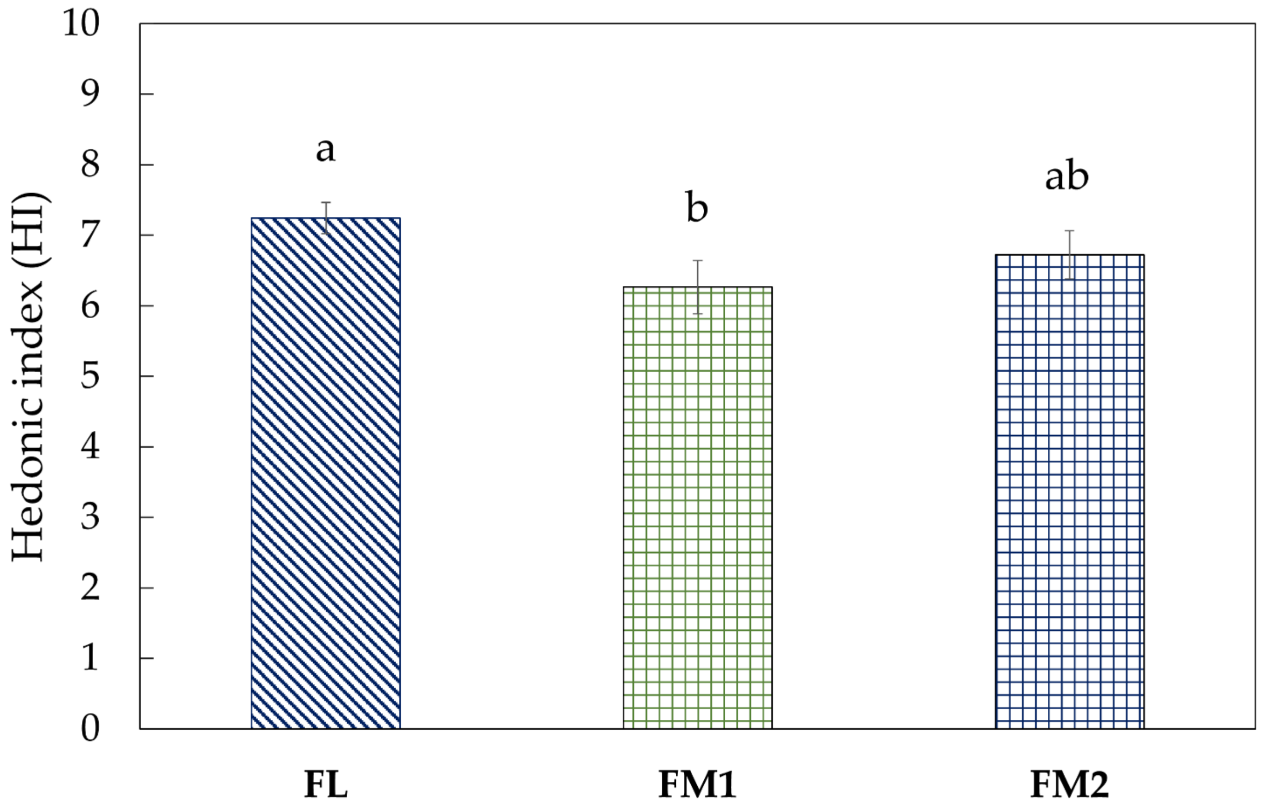

Figure S5. Hedonic index (HI) of the chestnut flour samples. Different letters indicate significant difference among values (Turkey HSD, $p < 0.05$)
